# Effect of physical activity in lymphocytes senescence burden in patients with COPD

**DOI:** 10.1152/ajplung.00151.2024

**Published:** 2024-08-06

**Authors:** Enrique Alfaro, Elena Díaz-García, Sara García-Tovar, Raúl Galera, Raquel Casitas, Elisabet Martínez-Cerón, María Torres-Vargas, José M. Padilla, Cristina López-Fernández, Paula Pérez-Moreno, Francisco García-Río, Carolina Cubillos-Zapata

**Affiliations:** ^1^Respiratory Diseases Group, Respiratory Service, La Paz University Hospital, IdiPAZ, Madrid, Spain; ^2^Biomedical Research Networking Centre on Respiratory Diseases (CIBERES), Madrid, Spain; ^3^Faculty of Medicine, Autonomous University of Madrid, Madrid, Spain; ^4^Pneumology Service, Hospital Universitario Clínico San Carlos, Madrid, Spain

**Keywords:** CD28, cellular senescence, chronic obstructive pulmonary disease, physical activity intervention, T lymphocytes

## Abstract

Chronic obstructive pulmonary disease (COPD) is regarded as an accelerated-age disease in which chronic inflammation, maladaptive immune responses, and senescence cell burden coexist. Accordingly, cellular senescence has emerged as a potential mechanism involved in COPD pathophysiology. In this study, 25 stable patients with COPD underwent a daily physical activity promotion program for 6 mo. We reported that increase of physical activity was related to a reduction of the senescent cell burden in circulating lymphocytes of patients with COPD. Senescent T-lymphocyte population, characterized by absence of surface expression of CD28, was reduced after physical activity intervention, and the reduction was associated to the increase of physical activity level. In addition, the mRNA expression of cyclin-dependent kinase inhibitors, a hallmark of cell senescence, was reduced and, in accordance, the proliferative capacity of lymphocytes was improved postintervention. Moreover, we observed an increase in functionality in T cells from patients after intervention, including improved markers of activation, enhanced cytotoxicity, and altered cytokine secretions in response to viral challenge. Lastly, physical activity intervention reduced the potential of lymphocytes’ secretome to induce senescence in human primary fibroblasts. In conclusion, our study provides, for the first time, evidence of the potential of physical activity intervention in patients with COPD to reduce the senescent burden in circulating immune cells.

**NEW & NOTEWORTHY** For the first time, we identified in patients with COPD a relation between physical activity intervention with respiratory function improvement and cellular senescence burden in lymphocytes that improved the T cell functionality and proliferative capacity of patients. In addition, our experiments highlight the possible impact of T-cell senescence in other cell types which could be related to some of the clinical lung complications observed in COPD.

## Graphical abstract

**Figure:**
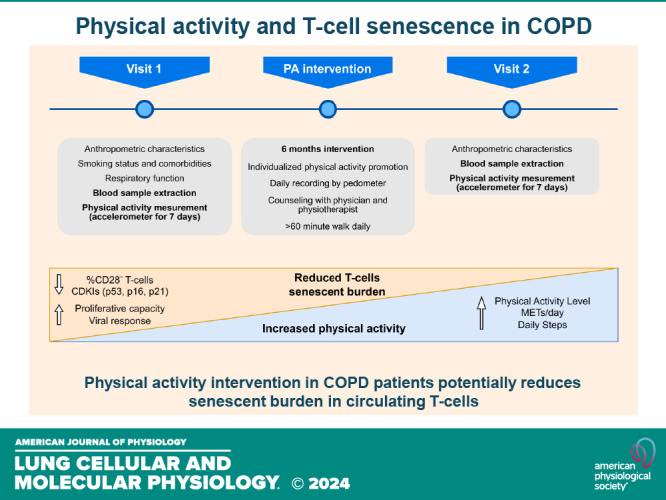

## INTRODUCTION

Chronic obstructive pulmonary disease (COPD) is the third leading cause of death worldwide and, by 2050, it is estimated to directly affect 500 million people (1). Long-term tobacco exposure, air pollution, and genetic background are considered the main causes of COPD, which is characterized by a persistent and irreversible airflow limitation (2). Despite decades of research, there is neither consensus on the mechanistic bases driving COPD disease, nor a complete explanation for the observed phenotypes. In the past years, the hypothesis that cellular senescence may play a major role in COPD pathophysiology is promising (3).

Patients with COPD suffer a deterioration of the respiratory and physical function in a process that implicates chronic pulmonary inflammation concomitantly with accumulation of senescent cells in the lung (3, 4) which has been related to the development of emphysema (5). Senescent cells are characterized by a terminal state of differentiation that includes abrogated proliferative capacity, apoptosis evasion, and metabolic and morphological changes (6). These cells are essential in controlling processes such as tissue repair and remodeling; however, their excessive accumulation imposes detrimental effects (7). In addition to respiratory impairment, COPD is characterized by a defective immune response which, at a cellular and molecular level, has been previously described as an “accelerated-aging phenotype” (8). An increase in senescent T lymphocytes has been reported in patients with COPD (9), which has been directly related to deregulated cytotoxic response and impaired overall response to infectious challenges (10). Senescent T cells, characterized by a loss of expression of costimulatory receptor CD28, accumulate through aging by T-cell clonal expansion due to accumulative activation which can be related to the chronic inflammatory process observed in patients with COPD. CD28^–^ T cells have been described as a reliable biomarker of an aging immune system and is related to inefficient immune response and chronic inflammatory diseases and aging-related conditions (11–13). In addition, senescent cells express a senescence-associated secretory phenotype (SASP) which includes the altered expression of cytokines and growth factors that may affect other tissues promoting further senescence burden and impairing correct tissue repair (14, 15).

Exercise is a promising intervention to improve the physical condition and reduce senescent cell burden in aging individuals as reported by Englund et al. (16). However, it is unclear how these interventions can benefit patients suffering age-associated and senescence-associated diseases, like COPD. In this line, several studies have highlighted the positive impact of physical activity intervention in respiratory parameters of patients with COPD including forced expiratory volume (FEV), forced vital capacity (FVC), and 6-min walk test as well as increased quality of life (17). Here, we hypothesize that an increase in daily physical activity in patients with COPD might be beneficial to their immune system by reducing senescent T-cell burden and increasing T cells functionality as well as modulating cytokine expression.

## MATERIALS AND METHODS

### Study Subjects

Patients with COPD were consecutively selected in the pulmonology service of the Hospital Universitario La Paz, Madrid, Spain. Subjects were older than 35 yr; clinical diagnosis of COPD and a postbronchodilator FEV_1_/FVC lower than the lower limit of normal; a postbronchodilator FEV_1_ lower than 70% of the predicted value; history of active smoking or former smokers with a cumulative tobacco consumption of at least 10 packs/yr; and optimized treatment according to current recommendations, with no changes in the last 8 wk. As exclusion criteria were considered: use of inhaled or systemic corticosteroids in the previous 3 mo; need for home oxygen therapy; a respiratory infection or exacerbation of another origin in the previous 2 mo; institutionalized patients; or clinical evidence of bronchial asthma, diffuse interstitial lung disease, thoracic or pleural box disease or neoplastic disease, as well as any locomotor or cognitive limitation that prevented ambulation. The study was approved by the research ethics committee of the Hospital Universitario La Paz (PI-2816) and all participants signed their informed consent.

Anthropometric characteristics were measured, including body composition (BF511 monitor, Omron Healthcare, Kyoto, Japan). Based on self-administered questionnaires and medical records, smoking status and comorbidities were recorded. All medications used by the participants at the time of the clinical evaluation were also listed.

Spirometry, body plethysmography, and measurement of diffusing capacity of the lungs for carbon monoxide (DLCO) were performed using a MasterScreen PFT system (Viasys, CareFusion, Würzbourg, Germany) equipped with the SentrySuite software, according to current standardization (18–20). Global Lung Initiative (GLI) equations were used as reference values (21–23) and tests were interpreted according to ERS/ATS technical strategies (24).

Respiratory muscle strength was assessed by the maximal inspiratory mouth pressure (PImax), measured with the same equipment according to ERS/ATS guidelines (25). Hautmann equations were used as reference values (26), and values below the lower limit of normal were considered reduced. As an indicator of overall muscle strength, hand-grip strength was measured in the dominant hand with a TKK 5001 dynamometer (Takei, Niigata City, Japan) as described previously (27, 28).

### Intervention

Baseline evaluation was performed before intervention. All patients were included in an individualized daily physical activity promotion program consisting of medical education and motivational visits, with reinforcement of daily recording of daily physical activity using a pedometer ONWALK 100. Counseling was provided by the patients’ physicians and a physiotherapist, and each session lasted ∼30 min. Patients were advised to increase their level of physical activity and to walk at least 60 min/day and to record this in the diary provided, along with any information related to their clinical status.

### Daily Physical Activity Measurement

Before and at the end of the physical activity promotion program, daily physical activity was measured using a multisensory armband SenseWear pro 3 accelerometer (Body Media Inc., Pittsburgh, PA), which the patient wore on the nondominant arm for seven consecutive days (29). The armband was described previously in a validation study for patients with COPD (30). Briefly, it incorporates a biaxial accelerometer that records steps per day and physiological sensors of energy expenditure (31). The physical activity level (PAL) was calculated by dividing the total daily energy expenditure by whole night sleeping energy expenditure (32). A physical activity level ≥1.70 defines a moderately active person, 1.40–1.69 defines a predominantly sedentary person, and <1.40 defines a very sedentary person (29, 31, 33).

### Blood Extraction and Peripheral Blood Mononuclear Cells Isolation

Peripheral blood (18 mL) was collected by venipuncture into EDTA tubes and plasma and peripheral blood mononuclear cells (PBMCs) were isolated by gradient centrifuge using 10-mL Ficoll-Paque Plus (Amersham Biosciences-Amersham, UK). Sequentially, cells were either, cultured, phenotyped by flow cytometry, or frozen for mRNA extraction. PBMCs were layered in six-well plates with Roswell Park Memorial Institute (RPMI) 1640 medium (ThermoFisher Scientific-Waltham, MA) for 1 h to allow the adherence of monocytes to the plastic surface. Nonadherent cells were recovered and placed in 24-well plates with RPMI medium supplemented with 10% fetal bovine serum (FBS) (ThermoFisher Scientific) at 37°C and 5% CO_2_. Nonadherent cells were cultured for 16 h challenged by 2 µg/mL of Imovax Polio (combination of immunogenic, inactivated *Poliovirus* strains; Sanofi Pasteur S.A.) to induce T-cell response. After 16 h, cells were harvested for flow cytometry phenotyping and supernatant was collected, aliquoted, and frozen at –80°C.

### Human Primary Fibroblasts Culture

Human primary fibroblasts were obtained from skin biopsies of healthy donors and cultured in Dulbecco’s modified Eagle’s medium (DMEM) (ThermoFisher Scientific) supplemented with 10% FBS at 37°C and 5% CO_2_. Fibroblasts from three caucasian donors (2 males, age 60 and 49; and 1 female, age 53), with no clinical sign of respiratory or neoplasic disease, were obtained and used in this study incorporating two technical replicas of each in the experiments. Subsequent passing was performed using trypsin 0.02% and neutralized in an equal volume of FBS.

Fibroblasts on passage 8 were seeded in 24-well plates (30,000 fibroblast/well), where they rested for 16 h. Next, fibroblasts were treated with 10% supernatant of cultured PBMCs or with 10% Roswell Park Memorial Institute 1640 (RPMI) medium. Fibroblasts were then cultured for 48 h. Supernatant was recovered, and cells were harvested for β-galactosidase assay or frozen for mRNA extraction. In addition, *Mycobacterium* testing was performed regularly in the cell culture facility and all cell cultures were free of *Mycobacterium.*

### Flow Cytometry T-Cell Phenotyping

PBMCs were harvested, washed in PBS, and treated following a standard protocol using the Transcription Factor Buffer Set from BD Biosciences. Cells were labeled (30 min, 4°C) with specific antibodies: CD137-PE(BD Biosciences, #555956), CD28-PE (BD Biosciences, #555729), CD4-APC (Immunostep S.L.-Salamanca, Spain, #4 A-100T), CD4-PerCP (Immunostep, #4PP-100T), CD8-APC (BD Biosciences, #555369), and PF-FITC (Miltenyi Biotec-Bergisch Gladbach, Germany, #130-096-668). Cells were acquired by FACS-Calibur cytometer and data were analyzed using FlowJo vX.0.7 software (BD Biosciences).

### Evaluation of T-Cell Proliferation

Proliferative capacity of lymphocytes was evaluated using Cell Trace CFSE cell proliferation kit (ThermoFisher Scientific) following manufacturer’s instructions. Briefly, PBMCs are incubated for 20 min in presence of CFSE and are washed with PBS and seeded in culture medium without CFSE. Cells are cultured for 96 h in presence of 2.5 µg/mL of pokeweed lectin from *Phylotaca americana* (Sigma-Aldrich-Burlington, MA), or in the presence of viral stimulation (Imovax). Cells are harvested and acquired using FACS-Calibur cytometer [Becton Dickinson (BD) Biosciences-Franklin Lakes, NJ].

### Cytokine Concentration Quantification

IL-6 and IL-8 concentrations were quantified in supernatants from PBMCs by using BD Cytometric Bead Array (CBA), human IL-6 or IL-8 Flex-Sets (BD Biosciences) and FACS-Calibur cytometer. Data were analyzed by FCAP Array software (BD Biosciences).

### mRNA Expression Quantification

RNA was extracted from PBMCs or fibroblasts using high pure RNA isolation kit by Roche (Penzberg, Upper Bavaria, Germany), and 0.25 μg of RNA was retrotranscribed using High-Capacity cDNA Reverse Transcription kit (ThermoFisher Scientific). mRNA expression was quantified by RT-qPCR using NZY Supreme qPCR Green MasterMix (Nzytech-Lisboa, Portugal) and CFX96 Touch Real-Time PCR Detection System (Bio-Rad Laboratories-Hercules, CA). Results were normalized to 18S expression. Specific primers were synthethized by Eurofins Genomics Srl (Vimidrone, MI, Italy): CDKN1A (p21) F 5′-GAGGCCGGGATGAGTTGGGAGGAG-3′ and R 5′-CAGCCGGCGTTTGGAGTGGTAGAA-3′; TP53 (p53) F 5′-CCGCAGTCAGATCCTAGCG-3′ and R 5′-AATCATCCATTGCTTGGGACG-3′; CDKN2A (p16) F 5′-GGGTTTTCGTGGTTCACATCC-3′ and R 5′- CTAGACGCTGGCTCCTCAGTA-3′; 18S F 5′- CGGCGACGACCCATTCGAAC-3′ and R 5′- GAATCGAACCCTGATTCCCC-GTC-3′.

### β-Galactosidase Assay

Quantification of the activation of β-galactosidase was performed using CellEvent Senescent Green Flow Cytometry Assay Kit (ThermoFisher Scientific) following manufacturer’s instructions. Briefly, cells were fixated in 4% paraformaldehyde for 10 min and β-galactosidase substrate was added to the cells and incubated at 37°C 0%CO_2_ for 2 h. Cells were acquired by FACS-Calibur cytometer with an absorption/emission spectrum of 490 nm/514 nm and data were analyzed by FlowJo vX.0.7 software.

### Statistical Methods

Unless otherwise indicated, results are presented as mean ± standard deviation and compared using a paired two-tailed Student’s *t* test or two-way ANOVA. Correlations were analyzed by Pearson linear regression. Detailed information of statistical methods and number of replicates is presented in the figure legends. Statistical calculations were performed in GraphPad Prism v.8 (GraphPad Prism) and SPSS v.20 (IBM-Statistics). Alluvial plot was performed in R Studio using package “ggplot2” and “ggalluvial.” Partial least squares-discriminant analysis (PLS-DA) model was calculated in R-Studio software using “caret” package. Variable importance projection (VIP) scores were calculated with “vip” and “dplyr” packages and plotted with “ggplot2” and “ggalt” packages.

## RESULTS

### Baseline Characteristics of Patients and Physical Activity Promotion

**Figure 1.**
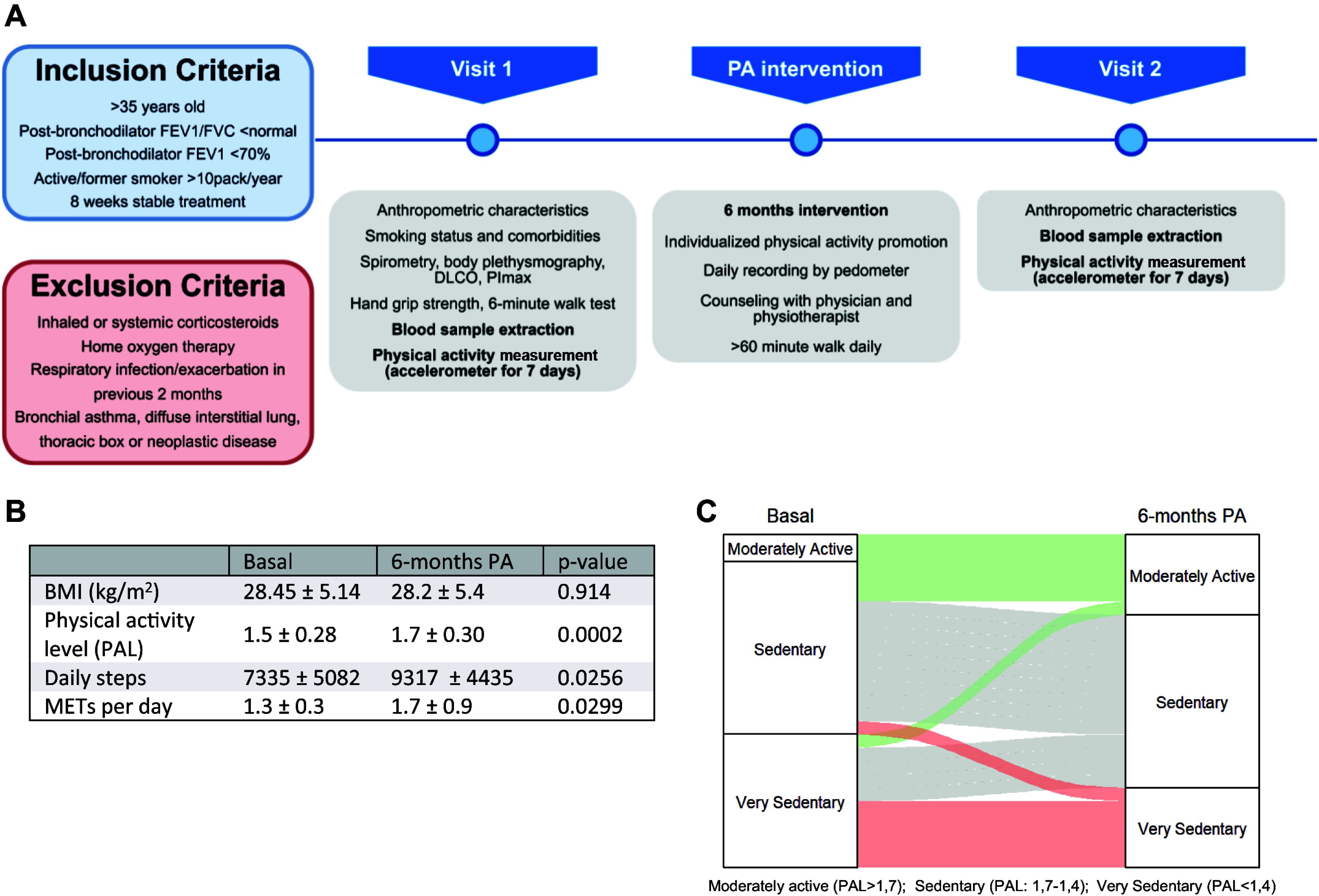
Physical activity measurements pre- and post-PA intervention. *A*: flowchart summarizing the study design: inclusion and exclusion criteria, measurements performed, and PA-intervention strategy are shown. *B*: basic physical characteristics of patients at baseline and after 6 mo of PA intervention. *n* = 25; results are means ± SD. Differences were analyzed by paired *t* test and *P* values are stated. *C*: alluvial plot of physical activity level (PAL) categorization before and after intervention. Moderately active patients (PAL > 1.7), sedentary patients (PAL: 1.7–1.4), very sedentary patients (PAL < 1.4). BMI, body mass index; DLCO, diffusing capacity of the lung for carbon monoxide; FEV1, forced expiratory volume in the first second; FVC, forced vital capacity; MET, metabolic equivalent of task; PA, physical activity; PAL, physical activity level; PImax, maximal inspiratory mouth pressure.

Twenty-five patients with COPD from Hospital Universitario La Paz, Madrid, Spain, clinically stable and with optimized treatment during the previous 3 mo, were recruited and baseline parameters were registered (Supplemental Table S1). Patients with COPD participated in a 6-mo individualized intervention to promote increased daily physical activity (PA) through a pedometer-stimulated program. Before and after intervention, daily physical activity was monitored for 7 days using a SenseWear armband tri-axial accelerometer, and blood samples were collected to assess the characteristics and functionality of circulating T lymphocytes (Fig. 1*A*). Patients increased their physical activity level (PAL), daily steeps, and metabolic equivalent tasks (METs) per day without significant changes in their body mass index (Fig. 1*B*). Most of the patients achieved a promotion of the daily PAL classification (29) suggesting a good adhesion to the intervention (Fig. 1*C*).

### T-Cell Senescence Burden Pre- and Postintervention

**Figure 2.**
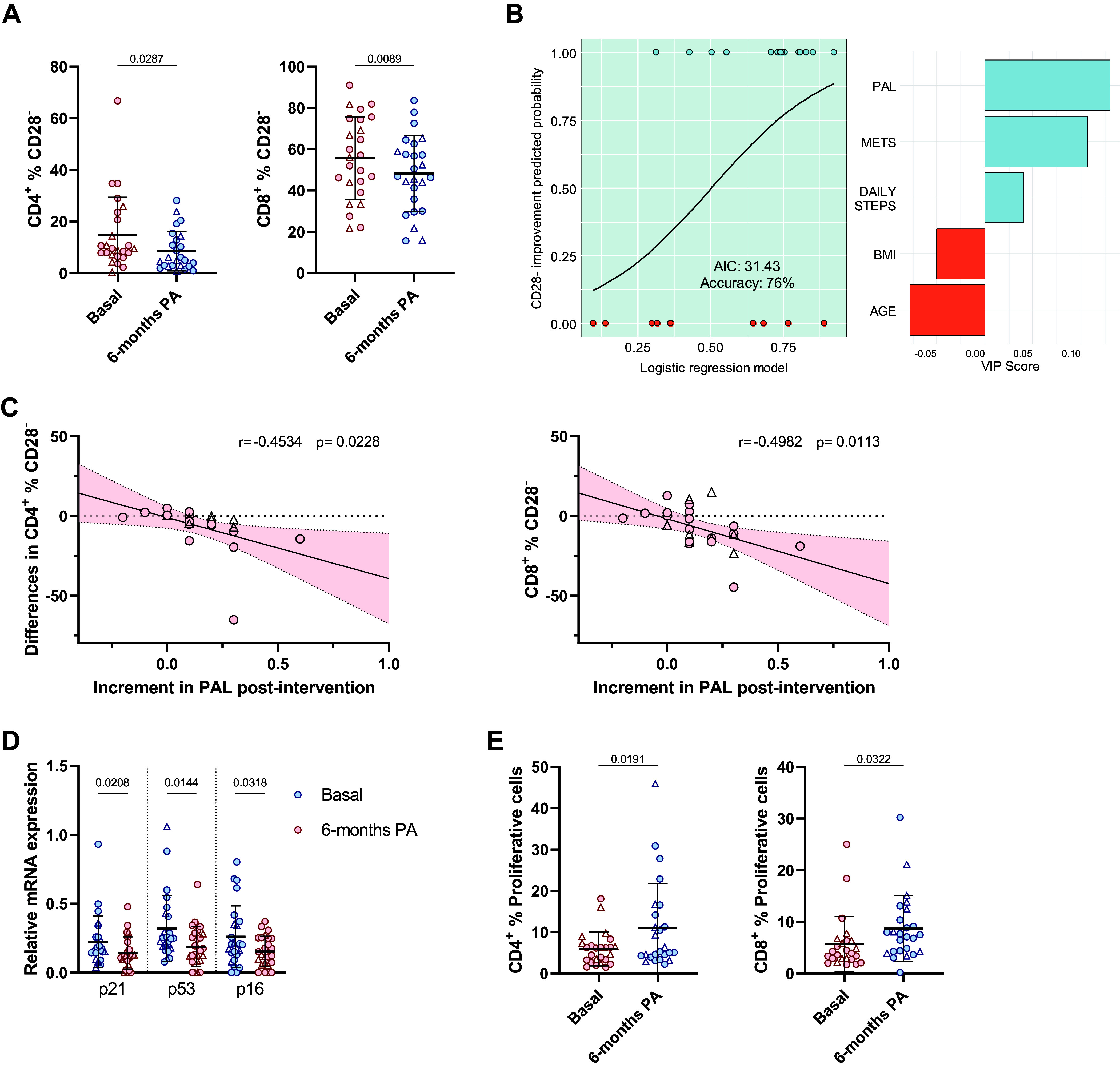
Lymphocyte senescence burden measurements pre- and post-PA intervention. *A*: comparison of the percentage of CD28^–^ T cells in the CD4^+^ and the CD8^+^ populations before and after intervention assessed by flow cytometry. *B*: logistic regression of PLS-DA scores integrating physical condition values and age at baseline to predict CD28^–^ percentage reduction after intervention (*left*) and VIP scores of variables included in the model (*right*). *C*: linear correlation between the increment of PAL after intervention and the difference in the percentage of CD28^–^ before and after intervention in CD4^+^ population (*left*) and CD8^+^ population (*right*). *D*: mRNA expression of CDKI (p21, p53, and p16) in PBMCs of patients with COPD before and after intervention. *E*: percentage of proliferative lymphocytes in CD4^+^ and CD8^+^ populations before and after intervention. *n* = 25; Results are means ± SD. Differences are analyzed by paired *t* test and *P* values are stated. In linear correlations, Pearson r and *P* value are annotated and shade represents 95% confidence interval. Triangles represent female subjects. PA, physical activity; PAL, physical activity level.

We determined the T-cell senescent burden by quantifying the accumulation of CD28^–^ T lymphocytes in peripheral blood of patients with COPD. PA intervention significantly reduced the percentage of CD28^–^ T cells (Fig. 2*A*), reducing the immunosenescent burden. Partial least-square discriminant analysis (PLS-DA) was performed to integrate physical condition values and age at baseline. PLS-DA scores differentiated patients who reduced the percentage of CD28^–^ T cells with an accuracy of 76%, and VIP scores showed PAL and METs per day as the most important variables affecting probability of CD28^–^ burden improvement (Fig. 2*B*). In accordance, the improvement in PAL correlated with the changes in percentage of CD28^–^ cells in CD4^+^ and CD8^+^ subsets after intervention (Fig. 2*C*). Another hallmark of cellular senescence is the expression of cyclin-dependent kinase inhibitors (CDKIs). We quantified the mRNA expression of CDKIs (p21, p53, and p16) in peripheral blood mononuclear cells (PBMCs) of patients with COPD and observed a reduction of these markers after 6 mo of PA intervention (Fig. 2*D*). CDKIs are involved in the cell cycle arrest which occurs in senescent cells. We observed an improvement in the proliferative capacity of COPD lymphocytes after PA intervention (Fig. 2*E*). Altogether, PA intervention improves patients’ physical parameters and may play a role in reducing senescent burden in patients with COPD in circulating T lymphocytes.

### T-Cell Function Is Restored after PA Intervention

**Figure 3.**
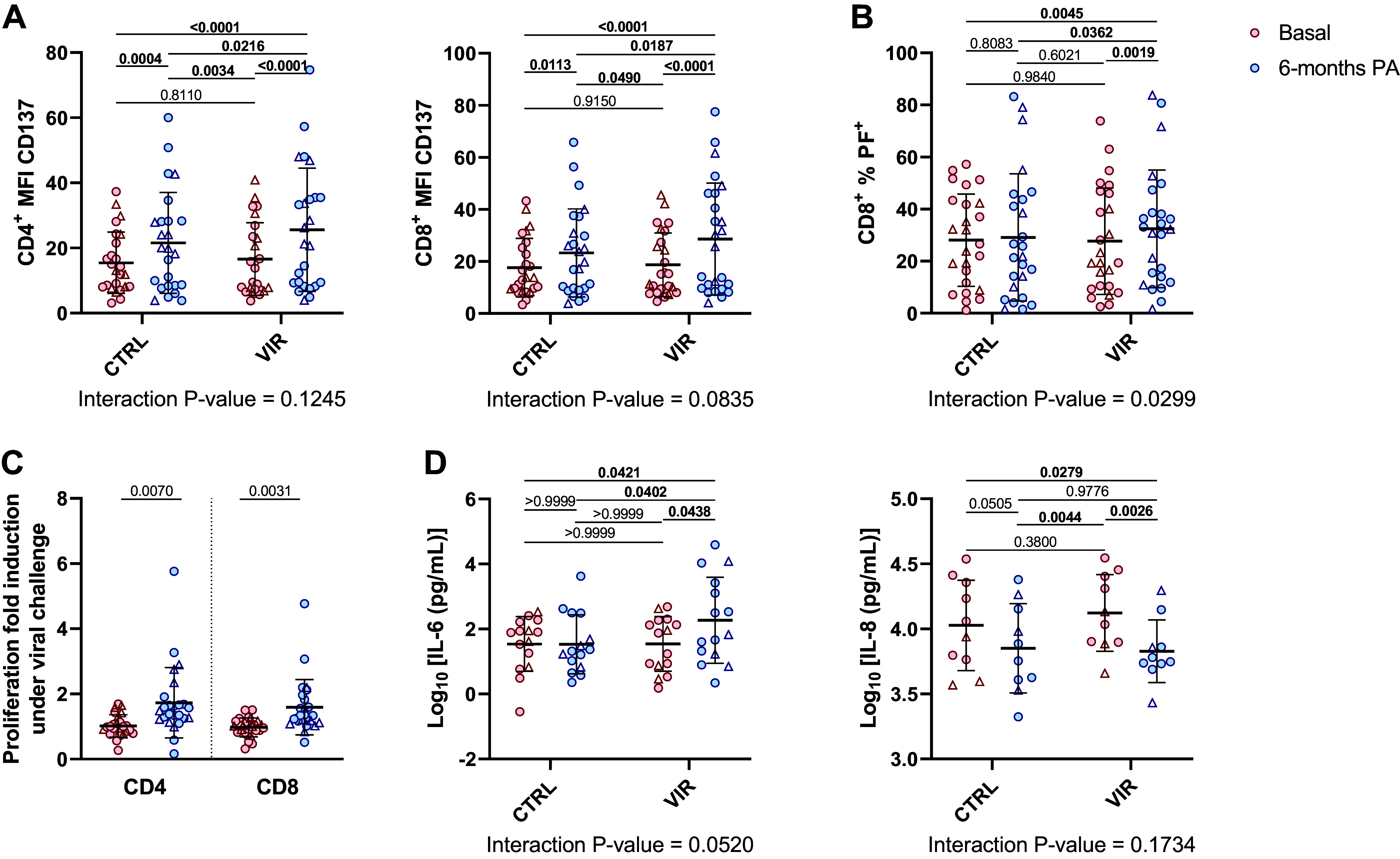
T-cell response to viral challenge. T cells from patients with COPD before (red) and after (blue) intervention are cultured for 16 h in RPMI medium (CTRL) or RPMI medium with viral stimulus (VIR). *A*: expression of CD137 in CD4^+^ and CD8^+^ lymphocytes measured by mean fluorescence intensity (MFI); *n* = 25. *B*: percentage of CD8^+^ cells expressing perforin (PF); *n* = 25. *C*: fold induction in percentage of proliferative CD4^+^ or CD8^+^ T cells under viral challenge; *n* = 25. *D*: concentration of IL-6 (*n* = 15) and IL-8 (*n* = 10) measured in supernatant from cultured T cells. Results are means ± SD. Differences are analyzed by two-way ANOVA with Tuckey’s multiple comparison tests and *P* values are stated. Differences in *C* are analyzed by paired *t* test and *P* values are stated. Triangles represent female subjects.

Viral infections represent a major threat to the lungs of patients with COPD and are involved in life-threatening events known as exacerbations. We explored the capacity of lymphocytes of patients with COPD to respond ex vivo to viral challenge before and after PA intervention. Lymphocytes extracted before and after the intervention were cultured 16 h in the presence of a viral challenge (inactivated *Poliovirus*). PA intervention was beneficial by increasing the expression of CD137 (Fig. 3*A*), an immune modulator that promotes T-cell response, proliferation, and survival. In addition, PA intervention improved the cytotoxic response of CD8^+^ T cells after viral challenge (Fig. 3*B*). Similar effect was observed regarding the proliferative capacity in response to viral challenge, only boosting proliferation in postintervention cells (Fig. 3*C*). Moreover, PA intervention modulated the response of T cells in terms of secreted cytokines by enhancing expression of IL-6 and reducing expression of IL-8 (Fig. 3*D*). Expression of SASP proteins by T cells is crucial as it can modulate the functionality of other tissues and cell types.

### COPD Lymphocytes Secretions Promote Fibroblasts Senescence

**Figure 4.**
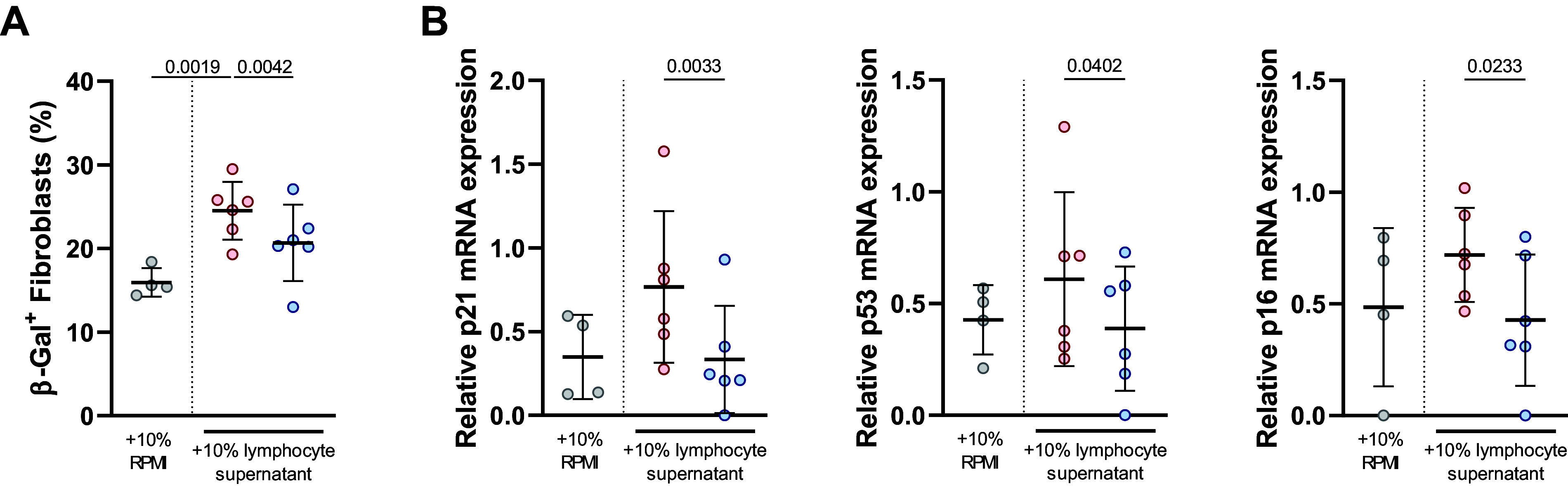
T-cell supernatants induce senescence in cultured healthy fibroblasts. Human primary fibroblasts treated for 48 h with 10% supernatant of lymphocyte culture from patients with COPD (*n* = 6) before (red) and after (blue) intervention or 10% RPMI medium as control (gray) (*n* = 4). *A*: percentage of fibroblast positive for β-galactosidase assay analyzed by flow cytometry. *B*: mRNA expression of CDKIs (p21, p53, and p21) in fibroblasts. Results are means ± SD. Differences are analyzed by *t* test and *P* values are stated.

Senescent lymphocytes may promote proinflammatory environment which can lead to further senescence. In this line, we recovered the supernatant of COPD lymphocyte culture and used it to stimulate healthy donor fibroblasts. Fibroblasts stimulated with supernatant from lymphocytes extracted during preintervention presented higher levels of β-galactosidase activity than fibroblasts stimulated with postintervention lymphocytes’ supernatant (Fig. 4*A*). In accordance, fibroblasts treated with preintervention lymphocyte supernatant expressed higher levels of CDKIs mRNA suggesting a more senescent phenotype (Fig. 4*B*). Altogether, postintervention lymphocytes’ supernatant retained less capacity to induce senescence in healthy fibroblasts, suggesting the capacity of PA intervention to modulate T-cell secretome and reduce its prosenescent potential in other tissues and cell types.

## DISCUSSION

In this study, stable patients with COPD underwent a physical activity promotion intervention for 6 mo. After intervention, patients improved their physical activity level concomitantly with reduced cellular senescence burden of T lymphocytes. COPD lymphocytes benefited from PA intervention by reducing their senescence markers and increasing their proliferative capacity and responsiveness to viral stimulation. In addition, an in vitro model with healthy fibroblasts showed PA intervention reduced the potential of COPD lymphocytes’ secretions to induce senescence in other cell types.

Previous studies have highlighted the possible implication of PA in reducing the number of senescent cells in different tissues and organs including skeletal muscle, vascular endothelium, and circulating leukocytes (34). Similar results were observed in this study suggesting the senotherapeutic potential of PA intervention in patients with COPD. Specifically, more physically active and younger patients of this cohort were more suited candidates for PA intervention to decrease T-cell senescence burden, which reinforces the importance of early changes in daily habits of patients with COPD. Immunosenescence in patients with COPD is a major issue as a deficient immune system in these patients may collaborate to maintain the chronic inflammation while simultaneously being defective in responding to infectious challenges. PA intervention in patients with COPD was beneficial to improve T lymphocytes functionality, reduce the expression of CDKIs which have been appointed as biomarkers of senescent cell burden and clinical biological age (35), and to improve T-cell proliferative capacity (36). Immune cell proliferation is essential to respond efficiently to infectious challenges (37) which in these patients are related to life-threatening events called exacerbations.

Immunosenescence is associated with a secretory phenotype that includes the release of cytokines, growth factors, and extracellular matrix remodeling molecules known as SASP (38). In this regard, PA intervention promoted the expression of IL-6 in response to viral challenge, which is a proinflammatory cytokine implicated in host defense, and limited the expression of IL-8. IL-8 is regarded as a SASP protein and may affect other tissues and induce further senescent burden (6). In addition, IL-8 is a chemoattractor of neutrophils and its expression in the lungs is related to enhanced neutrophil extravasation and has been associated with COPD exacerbations (39, 40). The dysregulation of the immune cells secretome by senescent processes can also alter tissue repair processes that concomitantly with chronic inflammation of the lungs can contribute to pulmonary lesions including emphysema. Investigating the possible role of lung-infiltrated senescent immune cells is the next step toward understanding their implications in COPD multiple phenotypes.

Physical activity intervention remains promising to reduce the immune senescent burden in patients with COPD although the molecular mechanisms involved remain mainly unclear. PA improves the overall fitness of patients, improving respiratory parameters (41) which are related to chronic inflammation (42) and may collaborate to reduce oxidative stress that is directly implicated in cell senescence (43). In addition, PA has been previously described to boost the antioxidant response of the body, increasing the endurance of cells and tissues to oxidative stress (44). Specifically, long-term exercise has been demonstrated to upregulate expression of protective and DNA repair proteins in circulating leukocytes (45). Eventually, PA intervention can be beneficial to the physical (41) and psychological (46) well-being of patients with COPD and, in this study, for first time, its impact on immune senescence has been assessed. Nevertheless, this study faces multiple limitations. First, no direct mechanism is investigated to relate PA intervention and senescent burden reduction. Second, the study focused on studying circulating lymphocytes and access to lung-infiltrated immune populations was not viable. Third, no follow-up of the patients has been performed yet. Fourth, in vitro experiments were performed with dermal fibroblasts and the impact on lung-derived cells is unexplored.

Understanding in detail the underlying cellular and molecular mechanisms by which PA intervention reduces senescent T-cell burden in patients with COPD is the next essential step. Furthermore, long-term follow-up studies are needed to unravel the clinical transcendence of these results mainly in terms of the immune system capabilities. PA intervention in patients with COPD is promising and improvements in immune system capabilities can benefit patients by reducing exacerbations or even cancer prevalence.

## DATA AVAILABILITY

The data supporting this study are available upon reasonable request to the corresponding author.

## SUPPLEMENTAL MATERIAL

10.6084/m9.figshare.26153302.v2 Supplemental Table S1: https://doi.org/10.6084/m9.figshare.26153302.v2.

## GRANTS

This study was supported by Instituto de Salud Carlos III (ISCIII) through the projects PI19/01612, PI22/01262, P2022/BMD-7224, Menarini to F. García-Río and COV20/00207, CP18/00028, PI19/01363 and PI22/01257 to C. Cubillos-Zapata; and co-funded by the European Union, Ayudas Luis Alvarez 2021 FIBHULP. C. López-Fernández was supported by Investigo technician fellowship from Comunidad Autónoma de Madrid (CAM).

## DISCLOSURES

No conflicts of interest, financial or otherwise, are declared by the authors.

## AUTHOR CONTRIBUTIONS

F.G.-R. and C.C.-Z. conceived and designed research; E.A., E.D.-G., S.G.-T., R.G., R.C., E.M.-C., M.T.-V., J.M.P., C.L.-F., P.P.-M., F.G.-R., and C.C.-Z., performed experiments; E.A., E.D.-G., R.G., R.C., E.M.-C., M.T.-V., J.M.P., P.P.-M., F.G.-R., and C.C.-Z. analyzed data; E.A., E.D.-G., P.P.-M., F.G.-R., and C.C.-Z. interpreted results of experiments; E.A., F.G.-R., and C.C.-Z. prepared figures; E.A., E.D., F.G.-R., and C.C.-Z. drafted manuscript; E.A., F.G.-R., and C.C.-Z. edited and revised manuscript; E.A., E.D.-G., S.G.-T., R.G., R.C., E.M.-C., M.T.-V., J.M.P., C.L.-F., P.P.-M., F.G.-R., and C.C.-Z. approved final version of manuscript.

## References

[1] Boers E, Barrett M, Su JG, Benjafield AV, Sinha S, Kaye L, Zar HJ, Vuong V, Tellez D, Gondalia R, Rice MB, Nunez CM, Wedzicha JA, Malhotra A. Global burden of chronic obstructive pulmonary disease through 2050. JAMA Netw Open 6: e2346598, 2023. doi:10.1001/jamanetworkopen.2023.46598. 38060225 PMC10704283

[2] Nussbaumer-Ochsner Y, Rabe KF. Systemic manifestations of COPD. Chest 139: 165–173, 2011. doi:10.1378/chest.10-1252. 21208876

[3] Chilosi M, Carloni A, Rossi A, Poletti V. Premature lung aging and cellular senescence in the pathogenesis of idiopathic pulmonary fibrosis and COPD/emphysema. Transl Res 162: 156–173, 2013. doi:10.1016/j.trsl.2013.06.004. 23831269

[4] Wrench CL, Baker JR, Monkley S, Fenwick PS, Murray L, Donnelly LE, Barnes PJ. Small airway fibroblasts from patients with chronic obstructive pulmonary disease exhibit cellular senescence. Am J Physiol Lung Cell Mol Physiol 326: L266–L279, 2024. doi:10.1152/ajplung.00419.2022. 38150543 PMC11281792

[5] Houssaini A, Breau M, Kebe K, Abid S, Marcos E, Lipskaia L, Rideau D, Parpaleix A, Huang J, Amsellem V, Vienney N, Validire P, Maitre B, Attwe A, Lukas C, Vindrieux D, Boczkowski J, Derumeaux G, Pende M, Bernard D, Meiners S, Adnot S. mTOR pathway activation drives lung cell senescence and emphysema. JCI Insight 3: e93203, 2018. doi:10.1172/jci.insight.93203. 29415880 PMC5821218

[6] Zavorsky GS, Hsia CC, Hughes JM, Borland CD, Guenard H, van der Lee I, Steenbruggen I, Naeije R, Cao J, Dinh-Xuan AT. Standardisation and application of the single-breath determination of nitric oxide uptake in the lung. Eur Respir J 49: 1600962, 2017. doi:10.1183/13993003.00962-2016. 28179436

[7] Woldhuis RR, de Vries M, Timens W, van den Berge M, Demaria M, Oliver BGG, Heijink IH, Brandsma CA. Link between increased cellular senescence and extracellular matrix changes in COPD. Am J Physiol Lung Cell Mol Physiol 319: L48–L60, 2020. doi:10.1152/ajplung.00028.2020. 32460521

[8] Sharma G, Hanania NA, Shim YM. The aging immune system and its relationship to the development of chronic obstructive pulmonary disease. Proc Am Thorac Soc 6: 573–580, 2009. doi:10.1513/pats.200904-022RM. 19934352 PMC5820858

[9] Savale L, Chaouat A, Bastuji-Garin S, Marcos E, Boyer L, Maitre B, Sarni M, Housset B, Weitzenblum E, Matrat M, Le Corvoisier P, Rideau D, Boczkowski J, Dubois-Rande JL, Chouaid C, Adnot S. Shortened telomeres in circulating leukocytes of patients with chronic obstructive pulmonary disease. Am J Respir Crit Care Med 179: 566–571, 2009. doi:10.1164/rccm.200809-1398OC. 19179485 PMC4850213

[10] Shivshankar P, Boyd AR, Le Saux CJ, Yeh IT, Orihuela CJ. Cellular senescence increases expression of bacterial ligands in the lungs and is positively correlated with increased susceptibility to pneumococcal pneumonia. Aging Cell 10: 798–806, 2011. doi:10.1111/j.1474-9726.2011.00720.x. 21615674 PMC3173515

[11] Guan Y, Cao M, Wu X, Yan J, Hao Y, Zhang C. CD28(null) T cells in aging and diseases: from biology to assessment and intervention. Int Immunopharmacol 131: 111807, 2024. doi:10.1016/j.intimp.2024.111807. 38471362

[12] Weng NP, Akbar AN, Goronzy J. CD28^−^ T cells: their role in the age-associated decline of immune function. Trends Immunol 30: 306–312, 2009. doi:10.1016/j.it.2009.03.013. 19540809 PMC2801888

[13] Vallejo AN. CD28 extinction in human T cells: altered functions and the program of T-cell senescence. Immunol Rev 205: 158–169, 2005. doi:10.1111/j.0105-2896.2005.00256.x. 15882352

[14] Martinez-Zamudio RI, Dewald HK, Vasilopoulos T, Gittens-Williams L, Fitzgerald-Bocarsly P, Herbig U. Senescence-associated beta-galactosidase reveals the abundance of senescent CD8+ T cells in aging humans. Aging Cell 20: e13344, 2021. doi:10.1111/acel.13344. 33939265 PMC8135084

[15] Pan XX, Wu F, Chen XH, Chen DR, Chen HJ, Kong LR, Ruan CC, Gao PJ. T-cell senescence accelerates angiotensin II-induced target organ damage. Cardiovasc Res 117: 271–283, 2021. doi:10.1093/cvr/cvaa032. 32049355

[16] Englund DA, Sakamoto AE, Fritsche CM, Heeren AA, Zhang X, Kotajarvi BR, Lecy DR, Yousefzadeh MJ, Schafer MJ, White TA, Atkinson EJ, LeBrasseur NK. Exercise reduces circulating biomarkers of cellular senescence in humans. Aging Cell 20: e13415, 2021. doi:10.1111/acel.13415. 34101960 PMC8282238

[17] Nymand SB, Hartmann JP, Ryrso CK, Rossen NS, Christensen RH, Iepsen UW, Berg RMG. Exercise adaptations in COPD: the pulmonary perspective. Am J Physiol Lung Cell Mol Physiol 323: L659–L666, 2022. doi:10.1152/ajplung.00549.2020. 36165500

[18] Graham BL, Steenbruggen I, Miller MR, Barjaktarevic IZ, Cooper BG, Hall GL, Hallstrand TS, Kaminsky DA, McCarthy K, McCormack MC, Oropez CE, Rosenfeld M, Stanojevic S, Swanney MP, Thompson BR. Standardization of Spirometry 2019 Update. An Official American Thoracic Society and European Respiratory Society Technical Statement. Am J Respir Crit Care Med 200: e70–e88, 2019. doi:10.1164/rccm.201908-1590ST. 31613151 PMC6794117

[19] Wanger J, Clausen JL, Coates A, Pedersen OF, Brusasco V, Burgos F, Casaburi R, Crapo R, Enright P, van der Grinten CP, Gustafsson P, Hankinson J, Jensen R, Johnson D, Macintyre N, McKay R, Miller MR, Navajas D, Pellegrino R, Viegi G. Standardisation of the measurement of lung volumes. Eur Respir J 26: 511–522, 2005. doi:10.1183/09031936.05.00035005. 16135736

[20] Graham BL, Brusasco V, Burgos F, Cooper BG, Jensen R, Kendrick A, MacIntyre NR, Thompson BR, Wanger J, Executive S. 2017 ERS/ATS standards for single-breath carbon monoxide uptake in the lung. Eur Respir J 49: 1600016, 2017. doi:10.1183/13993003.00016-2016. 28049168

[21] Quanjer PH, Stanojevic S, Cole TJ, Baur X, Hall GL, Culver BH, Enright PL, Hankinson JL, Ip MS, Zheng J, Stocks J; ERS Global Lung Function Initiative. Multi-ethnic reference values for spirometry for the 3-95-yr age range: the global lung function 2012 equations. Eur Respir J 40: 1324–1343, 2012. doi:10.1183/09031936.00080312. 22743675 PMC3786581

[22] Hall GL, Filipow N, Ruppel G, Okitika T, Thompson B, Kirkby J, Steenbruggen I, Cooper BG, Stanojevic S, contributing GLI Network members. Official ERS technical standard: Global Lung Function Initiative reference values for static lung volumes in individuals of European ancestry. Eur Respir J 57: 2000289, 2021. doi:10.1183/13993003.00289-2020. 33707167

[23] Stanojevic S, Graham B, Cooper B, Thompson B, Carter K, Francis R, Hall G; Global Lung Function Initiative (GLI) TLCO. Official ERS technical standards: Global Lung Function Initiative reference values for the carbon monoxide transfer factor for Caucasians. Eur Respir J 50: 1700010, 2017 [Erratum in Eur Respir J 56: 1750010, 2020]. doi:10.1183/13993003.00010-2017. 28893868

[24] Stanojevic S, Kaminsky DA, Miller MR, Thompson B, Aliverti A, Barjaktarevic I, Cooper BG, Culver B, Derom E, Hall GL, Hallstrand TS, Leuppi JD, MacIntyre N, McCormack M, Rosenfeld M, Swenson ER. ERS/ATS technical standard on interpretive strategies for routine lung function tests. Eur Respir J 60: 2101499, 2022. doi:10.1183/13993003.01499-2021. 34949706

[25] Laveneziana P, Albuquerque A, Aliverti A, Babb T, Barreiro E, Dres M, Dubé BP, Fauroux B, Gea J, Guenette JA, Hudson AL, Kabitz HJ, Laghi F, Langer D, Luo YM, Neder JA, O'Donnell D, Polkey MI, Rabinovich RA, Rossi A, Series F, Similowski T, Spengler CM, Vogiatzis I, Verges S. ERS statement on respiratory muscle testing at rest and during exercise. Eur Respir J 53: 1801214, 2019. doi:10.1183/13993003.01214-2018. 30956204

[26] Hautmann H, Hefele S, Schotten K, Huber RM. Maximal inspiratory mouth pressures (PIMAX) in healthy subjects–what is the lower limit of normal? Respir Med 94: 689–693, 2000. doi:10.1053/rmed.2000.0802. 10926341

[27] Wang YC, Bohannon RW, Li X, Sindhu B, Kapellusch J. Hand-grip strength: normative reference values and equations for individuals 18 to 85 years of age residing in the United States. J Orthop Sports Phys Ther 48: 685–693, 2018. doi:10.2519/jospt.2018.7851. 29792107

[28] Dodds RM, Syddall HE, Cooper R, Benzeval M, Deary IJ, Dennison EM, Der G, Gale CR, Inskip HM, Jagger C, Kirkwood TB, Lawlor DA, Robinson SM, Starr JM, Steptoe A, Tilling K, Kuh D, Cooper C, Sayer AA. Grip strength across the life course: normative data from twelve British studies. PLoS One 9: e113637, 2014. doi:10.1371/journal.pone.0113637. 25474696 PMC4256164

[29] Beeh KM, Watz H, Puente-Maestu L, de Teresa L, Jarreta D, Caracta C, Garcia Gil E, Magnussen H. Aclidinium improves exercise endurance, dyspnea, lung hyperinflation, and physical activity in patients with COPD: a randomized, placebo-controlled, crossover trial. BMC Pulm Med 14: 209, 2014. doi:10.1186/1471-2466-14-209. 25539654 PMC4364572

[30] Patel SA, Benzo RP, Slivka WA, Sciurba FC. Activity monitoring and energy expenditure in COPD patients: a validation study. COPD 4: 107–112, 2007. doi:10.1080/15412550701246658. 17530503 PMC3391963

[31] Watz H, Waschki B, Meyer T, Magnussen H. Physical activity in patients with COPD. Eur Respir J 33: 262–272, 2009 [Erratum in Eur Respir J 36: 462, 2010]. doi:10.1183/09031936.00024608. 19010994

[32] Watz H, Waschki B, Boehme C, Claussen M, Meyer T, Magnussen H. Extrapulmonary effects of chronic obstructive pulmonary disease on physical activity: a cross-sectional study. Am J Respir Crit Care Med 177: 743–751, 2008. doi:10.1164/rccm.200707-1011OC. 18048807

[33] Manini TM, Everhart JE, Patel KV, Schoeller DA, Colbert LH, Visser M, Tylavsky F, Bauer DC, Goodpaster BH, Harris TB. Daily activity energy expenditure and mortality among older adults. JAMA 296: 171–179, 2006. doi:10.1001/jama.296.2.171. 16835422

[34] Rossman MJ, Kaplon RE, Hill SD, McNamara MN, Santos-Parker JR, Pierce GL, Seals DR, Donato AJ. Endothelial cell senescence with aging in healthy humans: prevention by habitual exercise and relation to vascular endothelial function. Am J Physiol Heart Circ Physiol 313: H890–H895, 2017. doi:10.1152/ajpheart.00416.2017. 28971843 PMC5792201

[35] Liu Y, Sanoff HK, Cho H, Burd CE, Torrice C, Ibrahim JG, Thomas NE, Sharpless NE. Expression of p16(INK4a) in peripheral blood T-cells is a biomarker of human aging. Aging Cell 8: 439–448, 2009. doi:10.1111/j.1474-9726.2009.00489.x. 19485966 PMC2752333

[36] Fernandes JR, Marques da Silva CCB, da Silva AG, de Carvalho Pinto RM, da Silva Duarte AJ, Carvalho CR, Benard G. Effect of an exercise program on lymphocyte proliferative responses of COPD patients. Lung 196: 271–276, 2018. doi:10.1007/s00408-018-0107-9. 29525851

[37] Kalathil SG, Lugade AA, Pradhan V, Miller A, Parameswaran GI, Sethi S, Thanavala Y. T-regulatory cells and programmed death 1+ T cells contribute to effector T-cell dysfunction in patients with chronic obstructive pulmonary disease. Am J Respir Crit Care Med 190: 40–50, 2014. doi:10.1164/rccm.201312-2293OC. 24825462 PMC4226027

[38] Shrestha N, Chaturvedi P, Zhu X, Dee MJ, George V, Janney C, et al Immunotherapeutic approach to reduce senescent cells and alleviate senescence-associated secretory phenotype in mice. Aging Cell 22: e13806, 2023. doi:10.1111/acel.13806. 36967480 PMC10186597

[39] Wedzicha JA, Seemungal TA. COPD exacerbations: defining their cause and prevention. Lancet 370: 786–796, 2007. doi:10.1016/S0140-6736(07)61382-8. 17765528 PMC7134993

[40] Papi A, Luppi F, Franco F, Fabbri LM. Pathophysiology of exacerbations of chronic obstructive pulmonary disease. Proc Am Thorac Soc 3: 245–251, 2006. doi:10.1513/pats.200512-125SF. 16636093

[41] Mantoani LC, Rubio N, McKinstry B, MacNee W, Rabinovich RA. Interventions to modify physical activity in patients with COPD: a systematic review. Eur Respir J 48: 69–81, 2016. doi:10.1183/13993003.01744-2015. 27103381

[42] Barnes PJ, Baker J, Donnelly LE. Cellular senescence as a mechanism and target in chronic lung diseases. Am J Respir Crit Care Med 200: 556–564, 2019. doi:10.1164/rccm.201810-1975TR. 30860857

[43] Gambino V, De Michele G, Venezia O, Migliaccio P, Dall'Olio V, Bernard L, Minardi SP, Della Fazia MA, Bartoli D, Servillo G, Alcalay M, Luzi L, Giorgio M, Scrable H, Pelicci PG, Migliaccio E. Oxidative stress activates a specific p53 transcriptional response that regulates cellular senescence and aging. Aging Cell 12: 435–445, 2013 [Erratum in Aging Cell 18: e12962, 2019]. doi:10.1111/acel.12060. 23448364 PMC3709138

[44] Lanza IR, Short DK, Short KR, Raghavakaimal S, Basu R, Joyner MJ, McConnell JP, Nair KS. Endurance exercise as a countermeasure for aging. Diabetes 57: 2933–2942, 2008 [Erratum in Diabetes 61: 2653, 2012]. doi:10.2337/db08-0349. 18716044 PMC2570389

[45] Werner C, Furster T, Widmann T, Poss J, Roggia C, Hanhoun M, Scharhag J, Buchner N, Meyer T, Kindermann W, Haendeler J, Bohm M, Laufs U. Physical exercise prevents cellular senescence in circulating leukocytes and in the vessel wall. Circulation 120: 2438–2447, 2009. doi:10.1161/CIRCULATIONAHA.109.861005. 19948976

[46] Volpato E, Farver-Vestergaard I, Brighton LJ, Peters J, Verkleij M, Hutchinson A, Heijmans M, von Leupoldt A. Nonpharmacological management of psychological distress in people with COPD. Eur Respir Rev 32: 220170, 2023. doi:10.1183/16000617.0170-2022. 36948501 PMC10032611

